# Case Report: Coinheritance of Germline Mutations in *APC* and *BRCA1* in Colorectal Cancer

**DOI:** 10.3389/fonc.2021.658389

**Published:** 2021-03-25

**Authors:** Wei Huang, Jin Bian, Xiaoping Qian, Lin Shao, Haiyan Li, Lu Zhang, Lin Wang

**Affiliations:** ^1^Department of Oncology, Jiangsu Province Hospital of Chinese Medicine, Affiliated Hospital of Nanjing University of Chinese Medicine, Nanjing, China; ^2^Department of Oncology, Nanjing Jinling Hospital, Nanjing, China; ^3^The Comprehensive Cancer Centre, Nanjing Drum Tower Hospital, Medical School of Nanjing University, Nanjing, China; ^4^Department of Medicine, Burning Rock Biotech, Guangzhou, China; ^5^Department of Oncology, Nanjing Tongren Hospital, Nanjing, China

**Keywords:** familial adenomatous polyposis (FAP), *adenomatous polyposis coli* (*APC*) gene, *breast cancer susceptibility* (*BRCA*) gene, double germline mutations, next generation sequencing (NGS)

## Abstract

Deleterious mutations in *APC* gene cause the autosomal dominant familial adenomatous polyposis (FAP) which is typically characterized by the occurrence of hundreds to thousands of colorectal adenomas that eventually lead to colorectal cancers (CRCs). *BRCA1/2* are the two major susceptibility genes for breast and ovarian cancers. Here, we reported a coinheritance of mutations in *APC* and *BRCA1* genes in a 20-year-old CRC patient with typical clinical features for FAP. Multiple relatives in the family of the patient were affected by colorectal and other cancers. Next-generation sequencing analysis using a panel consisting of 53 hereditary cancer related genes revealed a maternally inherited *APC* (exon15cn_del) mutation and a paternally inherited *BRAC1* (p.lle1824AspfsX3) mutation. This is the first coexistence of *APC* and *BRCA1* mutations in a CRC patient with the mutation inheritance pattern comprehensively characterized in the family. The patient underwent a colonoscopy and a subtotal colectomy and was subsequently diagnosed with colonic adenocarcinomas accompanied with hundreds of tubulovillous adenomas. The case reveals the scenario where two disease-causing mutations of different hereditary tumor syndromes coexist, and illustrates the importance of evaluating detailed family history and performing a multiple-gene panel test in patients with hereditary cancer.

## Introduction

Colorectal cancer (CRC) is the third most common cancer worldwide (6.1%) and the second leading cause of cancer-related mortality (9.2%) (1). Approximately 10% of CRC patients are diagnosed at an age younger than 50 years, which is a hallmark of inherited cancer predisposition. Germline mutations in the mismatch repair genes *MLH1*, *MSH2*, *MSH6* and *PMS2*, or *EPCAM* lead to Lynch syndrome, the most common known cause of hereditary CRC and comprising 4% to 13.5% of early-onset CRC patients (2, 3).

*Adenomatous polyposis coli* (*APC*), located on chromosome 5q21-q22, is one of the tumor-suppressor genes frequently inactivated in the early progression of colorectal carcinogenesis (4, 5). Its primary transcript (NM_000038.6) has 16 exons with 1-15 coding a protein of 2843 amino acids (6). Deleterious germline mutations in *APC* cause familial adenomatous polyposis (FAP) which account for about 0.5% of all CRCs (4, 7). Individuals harboring a germline *APC* mutation can develop multiple adenomas caused by inactivation of the remaining allele in the colorectum *via* gain of additional somatic *APC* mutations or loss of heterozygosity (LOH) at this locus. FAP is typically characterized by the occurrence of hundreds to thousands of colorectal adenomas within 20 years which invariably lead to CRC if not detected early and removed.

Breast cancer susceptibility genes (*BRCA*) consisting of *BRCA1* and *BRCA2*, are also important tumor-suppressor genes (8, 9). *BRCA1* gene, located at chromosome 17q21, consists of 23 exons encoding a protein of 1863 amino acids. *BRCA2* is located at chromosome 13q12 and included 27 exons encoding a large protein product of 3418 amino acids. Mutations in *BRCA1/2* genes have been discovered in multiple malignancies (10). The cumulative breast cancer risk and ovarian cancer risk for mutation carriers are 5 times and 10-20 times higher than that for non-carriers (11), respectively. However, investigations on whether *BRCA1/2* mutations increase lifetime risk of developing colorectal cancer have yielded conflicting results (12–15).

In the present study, we describe a 20-year-old male with familial CRC harboring concurrent germline mutations in *APC* and *BRCA1* which were inherited maternally and paternally respectively.

## Case Report

**Figure 1:**
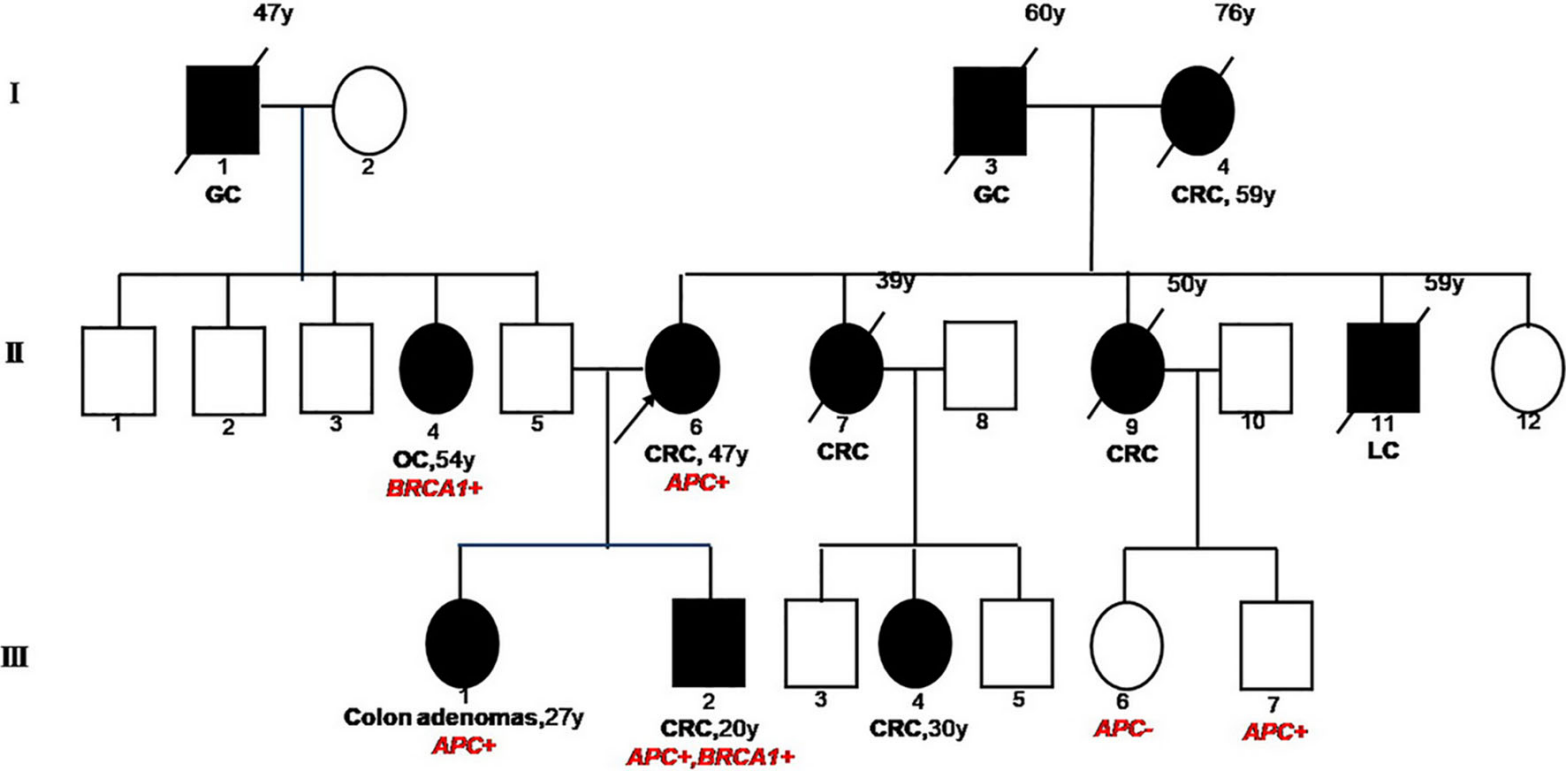
Familial Pedigree of case. The proband is indicated by an arrowhead. Squares represent males, circles represent females. Solid symbols represent affected individuals. Symbols with slash indicate deceased individuals. Age at cancer diagnosis is reported following the corresponding disease and the age of death is reported on the top right corner of symbol. GC, gastric cancer; CRC, colorectal cancer; OC, ovarian cancer; LC, lung cancer.

The reported patient was a 20-year-old male (**Figure 1**, III2) with a family history of cancers. His mother (II6) was first diagnosed with a grade II adenocarcinoma of colon accompanied with multiple polyps at the age of 48 and received a systemic chemotherapy. Subsequently, the detailed family history of his mother was assessed. His maternal grandfather (I3) died of gastric cancer when he was 60 years old. His maternal grandmother (I4) was diagnosed with CRC at the age of 59 and died at the age of 76. Two of his maternal aunts (II7&II9) died from CRC at the age of 39 and 50, respectively. His maternal uncle (II11) died of lung cancer at the age of 59. One of his female maternal cousins (III4) was diagnosed with CRC and subsequently received surgery at the age of 30 and was still alive.

**Figure 2:**
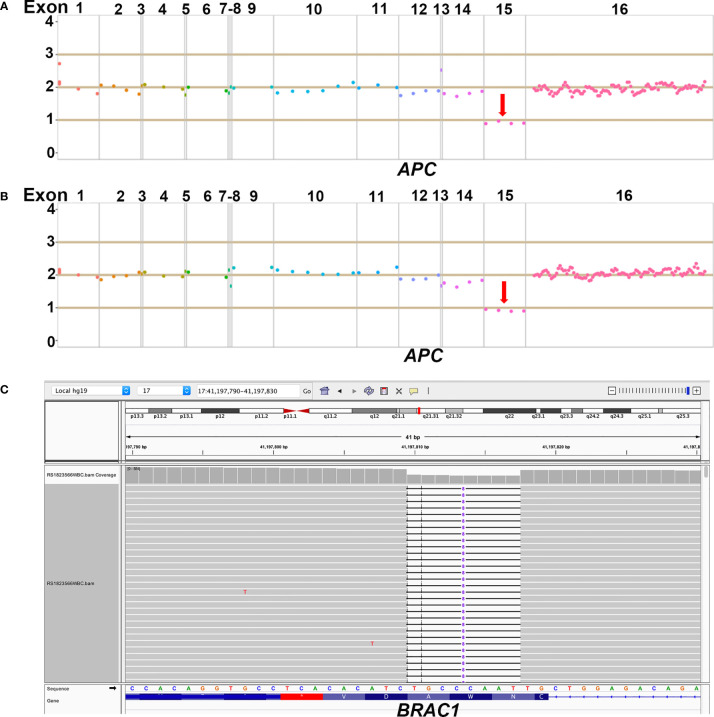
Demonstration of NGS results of *APC* and *BRCA1* germline mutations. **(A)** The heterozygous loss of exon 15 in *APC* gene was detected in the proband; **(B)** The same heterozygous loss of exon 15 in *APC* gene was also detected in the 20-year old male patient; **(C)** The heterozygous p.lle1824AspfsX3 in *BRCA1* gene was detected in the 20-year old male patient.

Due to the familial cancer history, in June 2018, the white blood cell sample was collected from the mother of the patient (proband, II6) and subjected to a next generation sequencing (NGS)-based genetic test using a panel consisting of 53 hereditary cancer related genes (Ugene, Burning Rock Biotech, China) with a median sequencing depth of 400x. The copy number variation analysis based on the sequencing depth revealed a copy number 1 for the exon 15 in *APC* gene, indicating a heterozygous loss of *APC* exon 15 (exon15cn_del, **Figure 2A**). Genetic test was also performed on two of the patient’s maternal cousins (III7&III6) and revealed the same *APC* mutation in the male cousin (III7) but no mutation present in female cousin (III6).

NGS with the same 53-gene panel performed on the reported patient (III2) and his sister (III1) revealed existence of the germline *APC* exon15cn_del mutation in both (**Figure 2B**). However, in addition to *APC* mutation, the male patient also harbored an open reading frame shift mutation in *BRAC1* (c. 5470_5477delATTGGGCA, p.lle1824AspfsX3, **Figure 2C**). The paternal family history of the patient showed that his paternal grandfather (I1) died from gastric cancer at the age of 47. His paternal aunt (II4) was diagnosed with ovarian cancer at the age of 54 years and is still alive. We also identified a same *BRAC1* (p.lle1824AspfsX3) germline mutation in her.

Taken together, the family clinical history and identified deleterious mutations were highly suggestive of FAP. The 20-year-old patient underwent a colonoscopy on Feb 21, 2019. More than ten polyps in size of 0.4-4cm with erosions on some of them were discovered. Three big polyps on the hepatic flexure of colon were biopsied which indicated high-grade intraepithelial neoplasia. The patient subsequently received a subtotal colectomy on March 25, 2019 and was diagnosed with stage T3N1bM0 colonic adenocarcinomas accompanied with more than one hundred of tubulovillous adenomas with the larger ones measuring 0.3-1cm. Immunohistochemical tests were performed with the surgical sample and revealed a status of HER2 (0), proficient DNA mismatch repair (pMMR) and BRAF (–). The patient subsequently received a modified oxaliplatin (L-OHP) with leucovorin (LV) and bolus/continuous infusion of 5-fluorouracil (5-FU) (mFOLFOX6) regimen for 12 cycles. Repeated image tests and a colonoscopy in Dec, 2019 revealed no evidence of recurrence or metastasis but the presence of multiple polyps measuring 0.3-0.8cm on the rectum, which were subsequently diagnosed with adenomatous polyps. The patient remained alive as the submission of the manuscript. The sister of the patient (III1) was also diagnosed with multiple adenomatous polyps of colon in May 2019 when she was 27 years and underwent subtotal colectomy.

## Discussion

Although this is the second report of CRC with concurrent *APC* and *BRCA* germline mutations, it is the first case which the inheritance of both mutations was well characterized by comprehensively sequencing the family members. Dolkar et al. first reported a 44-year-old Caucasian male with concurrent *APC* and *BRCA* germline mutations who had colonic adenocarcinoma accompanied with 15 additional colon polyps (16). The patient’s father had pancreatic cancer and his mother as well as his maternal cousin had colon cancer. Sequencing identified a pathogenic substitution mutation at nucleotide position 1213 in exon 9 of *APC* gene resulting in a premature stop codon (p.R405X) plus a deleterious c.8297delC variant in the *BRCA2* gene. The report only provided genetic tests for the proband therefore origins of mutations were not well recognized. In the present case, the male patient carried germline mutations in *APC* and *BRAC1* genes. By performing genetic analysis on multiple affected family members, we were able to delineate the inheritance pattern of mutations in this family. The mother of the patient (proband), sibling and maternal cousins all carried the *APC* mutation and his paternal aunt carried the *BRAC1* mutation which demonstrated a maternal origin of the former mutation and a paternal origin of the latter.

Pathogenic variants in *APC* gene are predominantly located in the exon 15 and always cause a premature truncation of the APC protein through nonsense substitutions or frameshifts (7). One of the mutations identified in this case was a heterozygous loss of exon 15 in *APC* gene which has been reported previously (17) and defined as a pathogenic variant according to the guidance of American College of Medical Genetics and Genomics (ACMG) (18).

BRCA proteins play essential roles in repair of DNA double-strand breaks *via* a homologous recombination mechanism (10). Deficiencies in BRCA proteins cause chromosomal instability which is associated with tumorigenesis. The open reading frame shift mutation in *BRAC1* (p.lle1824AspfsX3) reported in the case resulted in a truncated BRCA1 protein of 1825 amino acids. The mutation has been detected in 3 out of 133 Chinese women with familial breast/ovarian cancer and was characterized as pathogenic (19).

It is known that *BRCA1/2* are the two major susceptibility genes for breast and ovarian cancers. However it is still controversial whether the existence of germline mutation in *BRCA1/2* increases the risk of CRC. A study genotyped 2,398 CRC patients and 4,570 controls showed the presence of *BRCA1* mutation in 0.42% of cases and in 0.48% of controls (*P* = 0.8). Although the *BRCA1* mutation frequency was found slightly higher (0.93%) in patients with family CRC history, the study did not support the correlation of *BRCA1* mutations with increased risk of CRC (14). Another prospective study in 7,015 women with a *BRCA* mutation revealed an increased risk of CRC in carriers with *BRCA1* mutations younger than 50 years but not in carriers with *BRCA2* mutations or elder females (15).

Mutations in *APC* cause autosomal dominant FAP which often leads to CRC eventually. However, it is inconclusive whether *APC* and *BRCA* interact intrinsically which might predispose individuals with germline mutations in both genes to an increased risk of cancers. In the present case, the patient with double mutations developed colonic adenocarcinomas with hundreds of adenomas at the age of 20. He had a much earlier onset than other relatives in the family who only carried *APC* mutation. Previous studies investigating the coexistence of *APC* polymorphism I1307K with *BRCA* germline mutations demonstrated that *APC* I1307K increased the penetrance of *BRCA* mutations for breast cancer but not for ovarian cancer (20, 21). A study in mice also implied that although *APC* mutation might function early in the neoplastic process, coinheritance of a *BRCA2* alteration did not modify the *APC* mutation-driven phenotypes and therefore did not enhance tumorigenesis (22).

In present report, the well-specified family cancer history and the FAP-typical clinical characteristics observed in the proband made it relatively easy to uncover the disease-causing mutations. However in clinical practice, features of hereditary tumor syndrome are not always observed in patients and family histories are often not well-recognized. Therefore, the detection rate of pathogenic variants is always unsatisfactory. In a study aiming to screen the mutation rate in cancer susceptibility genes in 1,058 unselected CRC patients revealed 9.9% of patients carried mutations in cancer susceptibility genes and 7.0% carried mutations in non-Lynch syndrome (LS) genes. Notably, 15 of 23 carriers of high-penetrance non-LS mutations lacked classic clinical histories suggesting genetic factors that underlie CRC frequently occur beyond well-recognized familial CRC syndromes (23). Therefore, multigene panel testing in unselected patients with CRC will identify substantially more disease-causing mutations and bring more opportunities for genetically driven cancer prevention (3, 23).

The reported case harbors both *APC* and *BRAC1* pathogenic mutations which also confer an increased risk for duodenal cancer, pancreatic cancer, thyroid cancer, breast cancer and prostate cancer (7, 24). In order to detect tumor at early stage and receive proper management, the patient is suggested to consider routine esophagogastroduodenoscopy, thyroid and breast ultrasound imaging, pancreatic and prostate CT scan annually.

In conclusion, we reported a familial CRC case with coinheritance of mutations in both *APC* and *BRCA1* and well-characterized inheritance pattern in the family. The patient benefited from colonoscopy and subsequent management on the basis of genetic testing results. The case illustrates the importance of evaluating detailed family history and performing a multiple-gene panel test in cancer patient allowing for the identification of more disease-causing mutations and bringing more opportunities for genetically driven cancer prevention. In addition, one should also be aware of the scenario where double disease-causing mutations of different hereditary tumor syndromes coexist, in which the predisposition to specific cancer needs further investigation.

## Data Availability Statement

The original contributions presented in the study are included in the article/supplementary material. Further inquiries can be directed to the corresponding author.

## Ethics Statement

Written informed consent was obtained from the individual(s) for the publication of any potentially identifiable images or data included in this article.

## Author Contributions

LW: conception and design. WH: manuscript writing and manuscript review. WH and JB: clinical management of the patient. LS and HL: gene sequencing. XQ, LZ, and LW: manuscript revision. All authors contributed to the article and approved the submitted version.

## Conflict of Interest

Author LS, HL and LZ were employed by company Burning Rock Biotech.

The remaining authors declare that the research was conducted in the absence of any commercial or financial relationships that could be construed as a potential conflict of interest.
